# Association between procalcitonin levels and duration of mechanical ventilation in COVID-19 patients

**DOI:** 10.1371/journal.pone.0239174

**Published:** 2020-09-18

**Authors:** Martin Krause, David J. Douin, Timothy T. Tran, Ana Fernandez-Bustamante, Muhammad Aftab, Karsten Bartels

**Affiliations:** 1 Department of Anesthesiology, University of Colorado, School of Medicine, Aurora, Colorado, United States of America; 2 Department of Surgery, University of Colorado, School of Medicine, Aurora, Colorado, United States of America; University of South Carolina, UNITED STATES

## Abstract

**Background:**

Patients diagnosed with COVID-19 frequently require mechanical ventilation. Knowledge of laboratory tests associated with the prolonged need for mechanical ventilation may guide resource allocation. We hypothesized that an elevated plasma procalcitonin level (>0.1 ng/ml) would be associated with the duration of invasive mechanical ventilation.

**Methods:**

Patients diagnosed with COVID-19, who were admitted to any of our health system’s hospitals between March 9^th^-April 20^th^, 2020 and required invasive mechanical ventilation, were eligible for this observational cohort study. Demographics, comorbidities, components of the Sequential Organ Failure Assessment score, and procalcitonin levels on admission were obtained from the electronic health record. The primary outcome was the duration of mechanical ventilation; secondary outcomes included 28-day mortality and time to intubation. Outcomes were assessed within the first 28 days of admission. Baseline demographics and comorbidities were summarized by descriptive statistics. Univariate comparisons were made using Pearson’s chi-square test for binary outcomes and Mann-Whitney U test for continuous outcomes. A multiple linear regression was fitted to assess the association between procalcitonin levels and the duration of mechanical ventilation.

**Results:**

Patients with an initial procalcitonin level >0.1 ng/ml required a significantly longer duration of mechanical ventilation than patients with a level of ≤0.1 ng/ml (p = 0.021) in the univariate analysis. There was no significant difference in 28-day mortality or time to intubation between the two groups. After adjusted analysis using multivariable linear regression, the duration of mechanical ventilation was, on average, 5.6 (p = 0.016) days longer in patients with an initial procalcitonin level >0.1 ng/ml.

**Conclusion:**

In this cohort of 93 mechanically ventilated COVID-19 patients, we found an association between an initial plasma procalcitonin level >0.1 ng/ml and the duration of mechanical ventilation. These findings may help to identify patients at risk for prolonged mechanical ventilation upon admission.

## Introduction

Since the initial outbreak in the Hubei Province of China in November 2019, severe acute respiratory syndrome coronavirus 2 (SARS-CoV-2) has spread around the world and was declared a pandemic by the World Health Organization [1, 2]. Based on observational studies from the epicenters of the pandemic in Wuhan, China, the Lombardy region in Italy, and the New York City area in the United States, a significant portion of patients diagnosed with coronavirus disease 2019 (COVID-19) were admitted to the intensive care unit (ICU) for ventilatory support: Between 17%-24% of hospitalized patients and up to 72% of patients admitted to the ICU have required invasive mechanical ventilation [2–5]. While most ventilated patients fulfill the Berlin Definition for acute hypoxemic respiratory distress syndrome, the unique and variable clinical course of COVID-19 has made it difficult to predict the disease’s progression [6, 7].

Laboratory values, including plasma C-reactive protein, D-dimer, lactate dehydrogenase, and procalcitonin, are often elevated in patients with COVID-19 who required ICU-admission, mechanical ventilation, or died [8–11]. Knowledge of laboratory tests associated with prolonged mechanical ventilation and mortality in a health care environment constrained by high demand and limited resources, especially regarding ventilatory support, is critical. In a critically ill COVID-19 cohort from a large United States healthcare system, we hypothesized that elevated plasma procalcitonin levels would be associated with a longer duration of mechanical ventilation.

## Materials and methods

Ethical approval was obtained from the Institutional Review Board (Colorado Multiple Institutional Review Board #20–0677) on April 3^rd^, 2020, and the requirement for informed consent was waived. Data were collected retrospectively for any events before April 3^rd^, and prospectively going forward. We followed the Strengthening the Reporting of Observational Studies in Epidemiology (STROBE) guidelines for reporting observational studies [12].

### Aim, design, and setting

We aimed to identify if plasma procalcitonin levels on admission are associated with the duration of mechanical ventilation (primary outcome), 28-day mortality, and time to intubation (secondary outcomes) in a cohort of COVID-19 patients requiring mechanical ventilation. We designed an observational cohort study using automated data collection and manual chart review from the Electronic Health Record (EHR) from 12 hospitals within a large United States health care system.

### Participants, covariates, and outcomes

**Fig 1:**
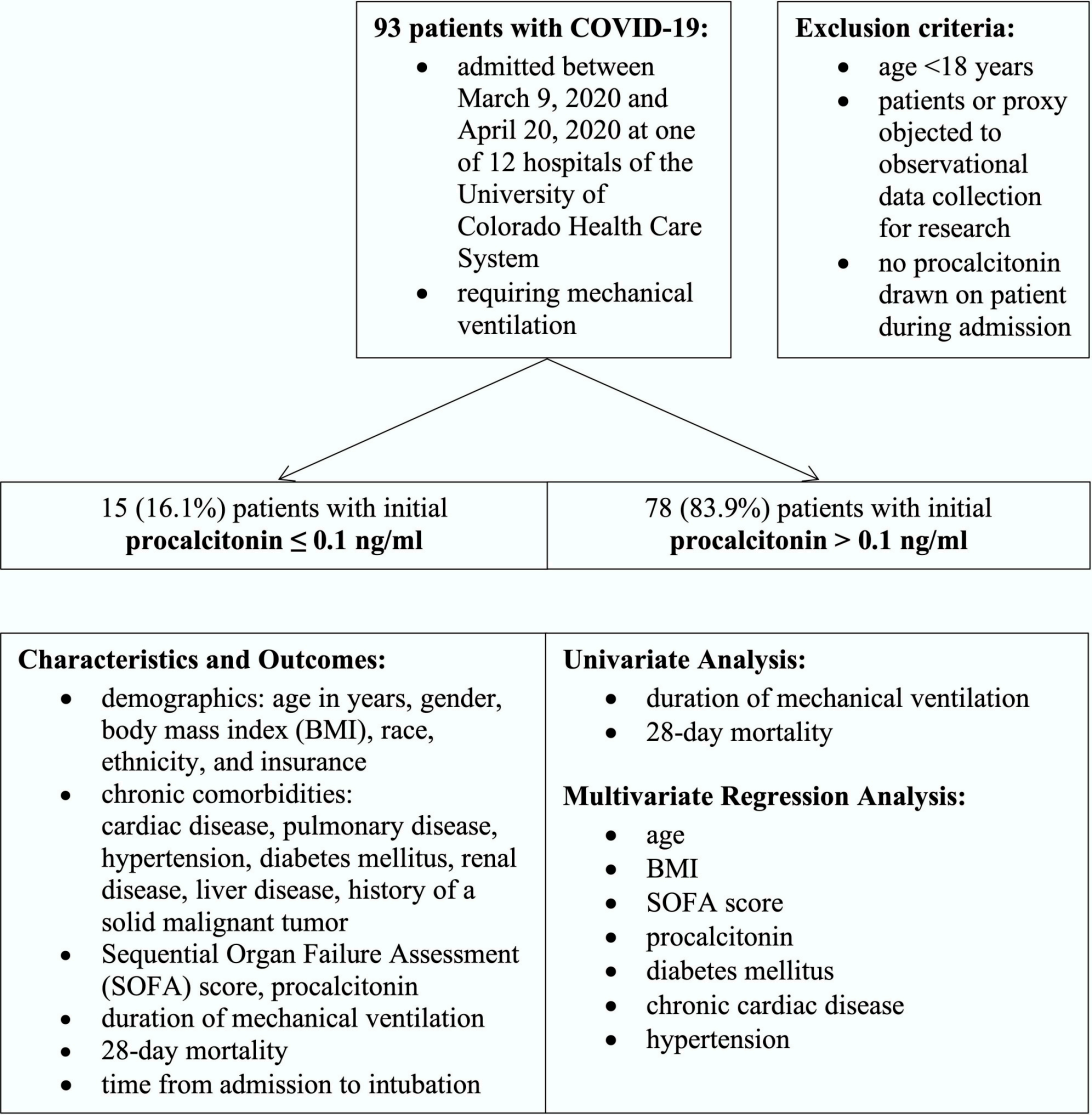
Flow-diagram of study cohort.

Patients with COVID-19 that were admitted between 03/09/2020 and 04/20/2020 to one of the health system’s ICUs requiring mechanical ventilation were eligible for inclusion in this study [13]. Exclusion criteria were age <18 years, patients’ or their proxies’ objection to observational data collection for research, or no procalcitonin level drawn during the admission (Fig 1).

Patient characteristics including relevant baseline demographics, chronic comorbidities, components of the Sequential Organ Failure Assessment (SOFA) score, and procalcitonin levels were obtained from the EHR. Demographics included: age in years, self-reported gender, body mass index (BMI) in kg/m^2^, race, ethnicity, and insurance status. We chose chronic comorbidities according to previously reported findings from our group [13] and others [10, 14] based on relevant pre-existing International Classification of Diseases codes (ICD-10) information from the EHR. Comorbidities were comprised of any history of cardiac disease, pulmonary disease, hypertension, diabetes mellitus, renal disease, liver disease, and solid malignancies. To calculate the SOFA score at hospital admission, we used the first drawn platelet count, creatinine, and bilirubin, the first recorded Glasgow Coma Scale score, and the lowest mean arterial pressure or the highest dose of vasoactive agents within the first eight hours of admission [15]. Furthermore, we obtained the first drawn procalcitonin level during the index admission. In-hospital mortality within 28 days was assessed based on the date of death as recorded in the EHR. Information on the duration of mechanical ventilation during the first 28 days of admission was obtained using manual chart review. Following published literature [16, 17], days were counted towards invasive mechanical ventilation, if the patient was mechanically ventilated within the first 28 days of admission, if a patient was reintubated within 48 hours (failure to extubate), or if a patient with a tracheostomy was on noninvasive ventilation or not requiring invasive/noninvasive ventilation for less than 48 hours (failure to wean). Furthermore, a patient who died within the first 28 days of admission was accounted for 28 days of invasive mechanical ventilation [16, 17]. The day of admission accounted for day 1 to assess time from admission to intubation and time from admission to death.

### Statistical analysis

Results were summarized using descriptive statistics, and continuous data were assessed for normality of distribution. Univariate comparisons were performed using Mann-Whitney U tests for continuous outcomes, and Pearson’s chi-square test for the binary mortality outcome. We then assessed if there was an association between an initial procalcitonin level of >0.1 ng/ml [18] vs. less and the duration of mechanical ventilation by fitting a linear regression model. Age, BMI, hypertension, diabetes mellitus, chronic cardiac disease, SOFA score on admission, and the first available procalcitonin level during the hospitalization were chosen as covariates in the model. Statistical analysis was performed using the software program SPSS, Version 26 (IBM Corporation, Armonk, NY).

A basic power analysis for a linear multiple regression model was performed using G*Power, version 3.1.9.2 [19]. Imputing an effect size f^2^ of 0.15, the number of tested predictors as 1, and the total number of predictors as 4, an alpha = 0.05 and a desired power of 90%, the sample size required would have been n = 73.

## Results

A total of 93 mechanically ventilated patients who tested positive for COVID-19 and had a procalcitonin level on file were included. The median duration of mechanical ventilation was 14 days. Patients’ demographics and comorbidities, SOFA scores on admission, initial procalcitonin levels, and outcomes are summarized in Table 1.

**Table 1:** Demographics, comorbidities, and outcomes.

Characteristic	Frequency
Total	93 (100)
Gender	
Men	62 (66.7)
Women	31(33.3)
Race	
Caucasian or White	44 (47.3)
African American or Black	23 (24.7)
Multiple races or other	26 (28.0)
Ethnicity	
Hispanic	31 (33.3)
Not Hispanic	62 (66.7)
Insurance status	
Managed care	33 (35.5)
Medicare	35 (37.6)
Medicaid, self pay, indigent, or other	25 (26.9)
Chronic cardiac disease	
Yes	41 (44.1)
No	52 (55.9)
Hypertension	
Yes	69 (74.2)
No	24 (25.8)
Chronic pulmonary disease	
Yes	39 (41.9)
No	54 (58.1)
Diabetes mellitus	
Yes	37 (39.8)
No	56 (60.2)
Preexisting renal disease	
Yes	28 (30.1)
No	65 (69.9)
Chronic liver disease	
Yes	18 (19.4)
No	75 (80.6)
History of solid malignant tumor	
Yes	11 (11.8)
No	82 (88.2)
Procalcitonin level >0.5 ng/ml	
Yes	25 (26.9)
No	68 (73.1)
Procalcitonin level >0.25 ng/ml	
Yes	42 (45.2)
No	51 (54.8)
Procalcitonin level >0.1 ng/ml	
Yes	78 (83.9)
No	15 (16.1)
**Characteristic**	**Mean**
Age–years	59 (15)
**Characteristic**	**Median**
Body mass index—kg/m^2^	30.5 (8.9)
SOFA score	4 (2)
**Outcomes**	**Median**
Days mechanically ventilated	14 (18)
Days from admission to intubation	2 (3)
**Outcomes**	**Frequency**
Mortality	
Yes	18 (19.4)
No	75 (80.6)

For categorical variables, column percentages are in parenthesis. For the continuous variable age, mean and standard deviation are reported; for the continuous variables body mass index (BMI), Sequential Organ Failure Assessment (SOFA) score, days on mechanical ventilation, and days from admission to intubation, median and interquartile range are reported. Days requiring mechanical ventilation and mortality were collected for 28 days post admission.

In the univariate analysis, patients with an initial procalcitonin level >0.1 ng/ml had a median duration of 17 days mechanical ventilation compared to patients with an initial procalcitonin level ≤0.1 ng/ml, who had a median duration of 10 days (p = 0.021) (Table 2). There was no significant difference in duration of mechanical ventilation for patients with a procalcitonin level >0.25 ng/ml vs. lower or >0.5 ng/ml vs. lower. There was also no significant difference in 28-day mortality or time from admission to intubation for patients with a procalcitonin level >0.1 ng/ml vs. lower (Table 2). In deceased patients with an initial procalcitonin level >0.1 ng/ml, death occurred at a median duration of 11 days.

**Table 2:** Univariate analysis.

Outcome	Procalcitonin ≤0.1 ng/ml n = 15	Procalcitonin >0.1 ng/ml n = 78	p-value
**Days mechanically ventilated, median (IQR)**	10 (5)	17 (17)	0.021
**Mortality, n (%)**	1 (6.7)	17 (21.8)	0.287
**Days from admission to intubation, median (IQR)**	2 (4)	2 (2)	0.692

The p-values signify exact two-sided p-values from Mann-Whitney U test for duration of mechanical ventilation and time from admission to intubation, and Chi-square test result for mortality; n: number of subjects, IQR: interquartile range, %: column percentages. Days requiring mechanical ventilation and mortality were collected for 28 days post admission.

The results from a fitted multivariable linear regression model with the duration of mechanical ventilation in days as the dependent variable are presented in Table 3. After adjustment for age, BMI, chronic cardiac disease, diabetes mellitus, hypertension, and SOFA score on admission, the multivariable linear regression model demonstrated that the duration of mechanical ventilation was on average 5.6 days (95% confidence interval 1.09–10.17, p = 0.016) longer in patients with an initial procalcitonin level >0.1 ng/ml compared to those with a lower procalcitonin level. In addition, older age (B = 0.22, 95% confidence interval 0.10–0.35, p = 0.001) and higher BMI (B = 0.23, 95% confidence interval 0.02–0.43, p = 0.033) were associated with a longer duration of mechanical ventilation. The other independent variables included in the regression model were not significantly associated with the duration of mechanical ventilation.

**Table 3:** Linear regression analysis for days mechanically ventilated as the dependent variable.

Characteristic	B coefficient	95% confidence limit	p-value
Intercept	-7.04	-19.45, 5.37	0.263
Age (years)	0.22	0.10, 0.35	0.001
Body mass index (kg/m^2^)	0.23	0.02, 0.43	0.033
Chronic cardiac disease (y/n)	-2.12	-5.73, 1.50	0.247
Diabetes mellitus (y/n)	1.29	-2.38, 4.95	0.487
Hypertension (y/n)	-1.04	-5.29, 3.22	0.630
Sequential Organ Failure Assessment score	-0.05	-1.05, 0.95	0.921
Procalcitonin >0.1 ng/ml (y/n)	5.63	1.09, 10.17	0.016

Overall model significance was p = 0.006 with seven degrees of freedom.

## Discussion

In the univariate analysis of our observational cohort study, procalcitonin levels >0.1 ng/ml on admission were associated with prolonged mechanical ventilation in critically ill COVID-19 patients. The multiple linear regression model revealed that the duration of mechanical ventilation was, on average, 5.6 days longer in patients with an initial procalcitonin level >0.1 ng/ml. Our findings demonstrate an association between elevated procalcitonin levels on admission and duration of mechanical ventilation in patients with COVID-19, thereby affirming our hypothesis. Given a shortage of ventilators in epicenters of this pandemic and the association between limited resources and COVID-19 mortality, we view our findings as highly relevant [20, 21]. In addition, prolonged mechanical ventilation is associated with ICU-acquired weakness and neurocognitive decline, which both contribute to short- and long-term-mortality [22, 23].

Early studies from China identified an elevated procalcitonin level as a predictor of the severity of COVID-19 infections but did not specifically explore an association between procalcitonin and duration of mechanical ventilation [3, 9, 11]. Procalcitonin is currently used to predict treatment failure and mortality in lower respiratory infections [24]. However, its plasma level often remains within the normal range in non-complicated cases of COVID-19. It has, therefore, been suggested as a helpful early marker of bacterial superinfections and disease progression [25]. Indeed, mechanically ventilated COVID-19 patients are more likely to suffer from bacteremia [26]. Identifying patients with COVID-19 who are at risk for prolonged mechanical ventilation may assist clinicians in allocating scarce resources and aid in patients’ risk stratification.

Our multivariate regression analysis was adjusted for severity of disease by including the SOFA score, for predisposing characteristics by including age, BMI, diabetes mellitus, hypertension, and chronic cardiac disease, and for procalcitonin levels on admission based on our own preliminary findings and other literature [8–11, 13, 27–29]. Besides a procalcitonin level of >0.1 ng/ml, duration of mechanical ventilation was associated with age and BMI in our multivariate regression model. Indeed, early literature from China and Europe identified age and obesity as risk factors for invasive mechanical ventilation [2, 7, 14, 30]. We did not find a correlation between elevated procalcitonin levels and 28-day mortality. However, 17 of 18 patients who died (94.4%) showed an elevated procalcitonin level suggesting that appropriately powered retro- or prospective studies might be able to identify such an association.

### Limitations

Our study has multiple weaknesses: First, an observational cohort study is not designed as a randomized controlled trial, and residual confounding remains possible [31]. For example, exclusion of patients without a documented procalcitonin level may have induced a selection bias. However, only three patients were excluded due to the lack of laboratory testing. Furthermore, our findings may not be representative of other areas of the United States or other countries. Lastly, prolonged mechanical ventilation and in-hospital mortality were determined within 28 days of the patient’s admission. A later follow-up could potentially show significant associations between demographics, comorbidities, and laboratory values in either of our outcomes, which were not identified at 28 days. Given the need for additional timely information during this pandemic as well as previous work by others [16, 17], we chose 28 days as the cut-off time-point for this study.

## Conclusions

Our observational study including 93 critically ill COVID-19 patients requiring mechanical ventilation found an association between a procalcitonin value of >0.1 ng/ml on admission and the duration of mechanical ventilation. Procalcitonin could be used to risk-stratify mechanically ventilated COVID-19 patients.
